# On the Use of Cyclic Cryogenic Treatment to Improve the Properties of High-Speed Steel

**DOI:** 10.3390/ma17235998

**Published:** 2024-12-07

**Authors:** Paweł Pieśko, Jarosław Korpysa, Magdalena Zawada-Michałowska

**Affiliations:** Faculty of Mechanical Engineering, Lublin University of Technology, ul. Nadbystrzycka 38D, 20-618 Lublin, Poland; p.piesko@pollub.pl (P.P.); j.korpysa@pollub.pl (J.K.)

**Keywords:** cryogenic treatment, high-speed steel, cutting edge wear

## Abstract

Cryogenic treatment is a process of controlled gradual cooling of the workpiece to a temperature ranging from −60 °C to even below −190 °C, holding the workpiece at this temperature and then slowly reheating it to ambient temperature. According to the current state of knowledge, the purpose of cryogenic treatment is to reduce the concentration of retained austenite by transforming it into hard martensite under low-temperature treatment. The retained austenite reduction in steels results in improved hardness, impact strength, and wear resistance. This study involved conducting comparative tests of the hardness, tensile strength, and impact strength of high-speed steel samples with and without cryogenic treatment, which made it possible to determine the effect of cyclic cryogenic treatment on the properties of this steel. In addition to that, machining tests were conducted to assess the life of a cutting tool edge made from both cryogenic-treated and non-cryogenic-treated high-speed steel. Also, the austenite concentration in the samples was measured by X-ray diffraction. Obtained results confirmed that the cyclic cryogenic treatment enhanced all tested properties of the high-speed steel.

## 1. Introduction

Cryogenic treatment is a heat treatment method in which the temperature of a workpiece is slowly decreased; the workpiece is maintained at this temperature usually for several hours or longer and then slowly reheated to ambient temperature. The aim of this treatment is to induce beneficial changes in the structure of the material. Cryogenic treatment is usually performed immediately after quenching, and often these processes are carried out in alternating cycles [1,2]. The essence of applying cryogenic treatment for steels is to remove retained austenite by inducing phase transformation into the martensitic phase under low temperature. Martensite is much harder than retained austenite as well as more stable at room temperature, so cryogenic-treated parts are characterised by dimensional and geometric stability. This is particularly important for precision components, tools, or measuring instruments. In addition to that, the low-temperature treatment causes carbides to be released in the steel, which usually results in its higher hardness, yield strength, and resistance. Other benefits of cryogenic treatment include increased resistance to abrasive wear and reduced residual stresses in the material [3]. Depending on the cryogenic temperature, a distinction is made between shallow and deep cryogenic treatment. Although the temperature ranges for these cooling methods may vary in some publications, they are usually between −60 °C and −160 °C for shallow cryogenic treatment while deep cryogenic treatment is conducted at lower temperatures [4].

Study [5] described the results obtained for SKD 11 tool steel after cryogenic treatment at −20 °C and −175 °C for 5 h. The cooling resulted in the precipitation of secondary carbides, the amount of which increased with the temperature descent. The induced changes resulted in increased hardness and abrasion resistance. Gecu et al. [6] investigated shallow (−72 °C) and deep (−196 °C) cryogenic treatment of AISI H13 steel. The application of cryogenic treatment resulted in reducing retained austenite concentration and transforming it into martensite. A higher amount of retained austenite was transformed into martensite when a lower cryogenic temperature was applied. This resulted in increased hardness and abrasive wear resistance, but the application of an additional tempering operation reduced these properties again. H13 steel was also investigated in [7] by subjecting it to shallow (−80 °C) and deep (−185 °C) cryogenic treatment. The cryogenic treatment at −80 °C produced a significantly smaller amount of martensite compared to the lower-temperature treatment. Nevertheless, this amount was still higher than that obtained by quenching combined with tempering. The cryogenic treatment conducted at the two temperatures resulted in an approx. 20% and 30% reduction in the abrasive wear rate, respectively. In contrast, the highest hardness was achieved after quenching alone. The effects of different heat treatment methods for H13 steel were also compared in [8]. It was found that an additional tempering operation after the deep cooling process increased the hardness of the material. This was attributed to the smaller grain size and more uniform distribution of martensite and carbides, the formation of which was induced by cryogenic treatment. In addition to that, the thermal fatigue tests showed that both the density and the length of cracks in the treated samples were lower than in the non-treated samples.

A review of the literature [9,10,11] demonstrates that the results of cryogenic treatment are highly dependent on the accompanying thermal treatments applied to a given material. The most important ones include the type of heat treatment, its duration, and temperature. Parcianello et al. [12] demonstrated that the application of deep cooling at −196 °C made it possible to enhance the hardness of M2 steel, but additional tempering operations made the hardness decrease again. Higher hardness was observed for the tempered material without prior cooling. Nevertheless, the cryogenic-treated samples showed a lower abrasive wear rate. Similar correlations were also observed for cold work tool steels [13]. Following the cryogenic treatment at −196 °C, the hardness of the material increased, while further tempering resulted in a decrease in the hardness of the material. It was shown that the result of cryogenic treatment also depended on the way in which the material was manufactured [14]. A comparison of steels produced by casting and powder metallurgy showed that the latter materials exhibited a greater increase in hardness and an almost fourfold lower abrasive wear rate. This results from a higher quantity of martensite and a more uniform distribution of carbides in the powder steels.

Feng et al. [15] investigated the cryogenic treatment for GCr15 steel, showing that even though the treatment reduced the retained austenite concentration, the best effect was obtained by combining cooling and laser peening, as this resulted in a significant improvement in the hardness and elastic modulus of the material. This, however, resulted in an approximately sevenfold increase in the surface residual stresses, whereas after cooling, the increase was fourfold relative to the material without any treatment. In study [16], different heat treatment methods were applied to H13 steel. Similar tensile strengths were obtained for all treatment methods, and the use of deep cooling with tempering caused an almost twofold increase in elongation while the use of cooling alone reduced it. Study [17] showed that the selection of an appropriate cooling temperature was very important. The residual stresses were reduced by up to about 60% when the cryogenic treatment temperature was −130 °C. This is mainly due to the reduction in grain size, as no phase transformations were observed. The use of a higher (−80 °C) as well as a lower (−190 °C) cryogenic treatment temperature led to the formation of residual stresses that were higher than those in the samples subjected to ageing. Chen et al. [18] demonstrated that the effect of cryogenic treatment varied, depending on the holding time of the sample at ambient temperature after quenching. After a 2 h holding time, the samples had a higher yield strength and lower tensile strength, compared to 60-day holding time. Nevertheless, this had little effect on hardness and total elongation. The application of additional ageing led to an increase in both the yield stress and tensile strength. The change in the structure and properties of the material occurs not only during prolonged cooling at low temperatures, but also during short cooling of the material directly during strength tests [19,20]. Cryogenic treatment can also be a good complement to the gas–laser cutting method. It is a method that allows for precise cutting of elements. However, due to the large amount of emitted energy, a heat-affected zone is created, in which changes occur in the material structure. The main result is the formation of large amounts of retained austenite in close proximity to the cut surface [21,22,23]. The use of cryogenic treatment would therefore allow for reducing the amount of retained austenite.

Owing to its beneficial effects, such as improved hardness and strength, cryogenic treatment is used not only for workpieces, but also for cutting tools. This is particularly important in terms of reducing tool wear and prolonging tool life. Ozbek et al. [3] subjected coated carbide inserts to shallow and deep cryogenic treatment first and then used them for turning 41Cr4 steel. The study showed that the cryogenic-treated inserts exhibited improved properties due to higher concentrations of secondary carbides. Their hardness increased, reducing the tool wear indicator *VB* by up to about 18% for deep cryogenic treatment and by about 12% for shallow cryogenic treatment. The use of deep cryogenic treatment also reduced the value of the roughness parameter *Ra* by up to 20%.

In study [24], carbide inserts were cryogenic-treated at −196 °C. The results showed that the Eta phase was reduced in both the coated and the uncoated tool. The material structure change resulted in a longer tool life during turning EN24 steel. The uncoated tool had a 50% longer tool life after heat treatment. For the coated tools, the tool life increase was smaller; yet, it was almost 25% in favour of the cryogenic-treated tool. The cooling time of the material is also significant [25]. A comparison of carbide inserts cooled at −196 °C for 24 and 36 h showed an improvement in the milling performance of 450 stainless steel inserts relative to the inserts without cryogenic treatment. The application of cryogenic treatment led to reduced tool wear, lower cutting temperature and forces, and recued surface roughness. More favourable results were achieved by using a shorter cooling time for the cutting inserts. A different relationship was observed in [26], where AlTiN-coated carbide inserts cooled at −175 °C were used in the milling process of a 6061-T651 aluminium alloy. The results confirmed the increased hardness of the cryogenic-treated insert; however, the use of optimum machining parameters produced a more beneficial effect for the non-cryogenic-treated insert, resulting in lower surface roughness and wear.

An important factor affecting machining is tool wear, which occurs primarily in the contact zone between the tool and the machined surface, thus increasing the cutting force and temperature, and further accelerating the wear rate, which affects the machining process and its end result [27,28]. This effect is primarily related to the dimensional accuracy of the workpieces and their surface roughness. It can particularly be noticed in drilling, where the tool diameter reduction due to wear causes manufacturing errors [29,30,31]. This is also a very important aspect in micro-machining, where special requirements are imposed for the accuracy and manufacturing precision of workpieces [32,33,34]. Although tool wear depends on many factors, the key factor is workpiece material. It is most problematic in the machining of hard-to-machine materials such as titanium alloys [35,36,37] or nickel-based alloys [38,39,40]. For materials with much better machinability such as aluminium alloys [41,42,43] or magnesium alloys [44,45], tool wear is considerably lower and therefore does not play such a significant role in the machining process. However, its effect cannot be completely ignored.

The significance of the chemical composition of materials was confirmed by Hrechuck et al. [46], who studied tool wear depending on the inclusions in 316L steel. They demonstrated that the addition of Al_2_O_3_ particles and increased grain size caused a significant increase in wear due to the increased hardness of the material. For the inclusion-free steel, the resulting *VB* indicator was up to 60 percent lower than that obtained for steel with 15 μm particles. The inclusions also had impact on the shape of the wear scar. The cutting tool material, particularly its hardness, was found to be of vital importance, too. The wear was nearly twice lower when the turning process of 304 steel was conducted with a carbide tool than with a high-speed steel tool [47]. It was also shown that, for both cases, the wear increased significantly with increasing the cutting parameters. The effects of cutting conditions were also analysed in a study [48] related to a turning process for 316 L steel. Similarly, faster tool wear occurred when higher cutting parameters were used, especially the feed and depth of the cut. The use of minimum-quantity lubrication (MQL) had a significant effect on reducing tool wear. This resulted in a significant reduction in the coefficient of friction, also leading to reduced cutting forces and temperature.

The use of different cooling methods was also investigated in [49]. The turning process of AISI-1040 steel was carried out under dry conditions, with flood cooling, with MQL cooling, and with MQL cooling with the addition of nanoparticles (NF-MQL). The best result was obtained for the machining process conducted with NF-MQL cooling, yielding about 50% lower flank wear (*VB* indicator) and about 36% lower crater wear (*KT* indicator) relative to the dry processing. The lower wear resulted in reduced cutting temperature, vibration, and improved surface texture (reduced roughness). Tool wear also depends on the type of tool coating. Study [50] investigated the effects of turning 316 L steel using ceramic inserts coated with TiAlSiV5N and TiAlSiV11N. The use of tool coatings results in up to eightfold lower flank wear. It was also found that the wear of the coated inserts was also less dependent on cutting speed. This results from the lower coefficient of friction along with lower cutting temperature and force for the coated tools. Lower tool wear also led to improved surface roughness. The benefits of using tool coatings were also demonstrated in [51]. The use of coated inserts for turning X20Cr13 and X8CrNiS18-9 stainless steels resulted in an almost threefold increase in the cutting length for the Ti(C,N) + Al_2_O_3_ + TiN-coated samples and an almost fourfold increase in the cutting length for the TiN-coated sample. The wear also affected surface roughness. Despite similar results, the best surface quality was obtained with the TiN-coated insert.

Cryogenic treatment is also increasingly used in subtractive machining as one of the machining zone cooling methods. It is used to improve the cutting process by reducing the cutting temperature, which can have a negative effect on the tool and workpiece; e.g., it can lead to reduced surface quality. The use of cryogenic treatment also results in improved lubrication properties, as well as reduced friction [52,53,54]. The positive effects of cryogenic treatment have been observed in relation to turning as well as other machining processes [55,56].

## 2. Materials and Methods

**Figure 1:**
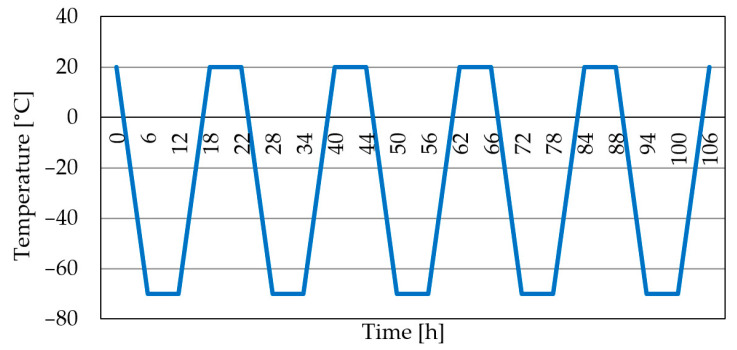
Cycle of temperature variations during cryogenic treatment.

As mentioned in the Introduction, the retained austenite reduction in hardened steels resulting from cryogenic treatment should lead to improvement of their mechanical properties, hardness, and abrasion resistance. Initially, M2 steel samples were subjected to shallow cryogenic treatment (−70 °C) for 6 h (one cycle), but the obtained retained austenite reduction was considered unsatisfactory. Based on the authors’ own experience, it was therefore decided that the cyclic cryogenic treatment would be conducted according to a scheme shown in Figure 1. The samples and tools were subjected to a total of five cooling cycles, each comprising the following phases:

- Temperature descent to −70 °C for 6 h;
- Cryogenic processing at −70 °C for 6 h;
- Temperature ascent to 20 °C for 6 h;
- Stabilisation at 20 °C for 4 h, and the repetition of the cycle.

A new proprietary solution used in the conducted research is cyclic cryogenic treatment. The treatment is usually carried out in one cycle, but based on the research results, it was found that such an approach gives positive effects. Additionally, the equipment did not require liquid nitrogen, which simplified the tests.

The cryogenic-treated and non-cryogenic-treated samples were subjected to the following:

- Diffractometric tests for retained austenite concentrations using a Theta-Theta Edge diffractometer (G.N.R. S.r.l, Agrate Conturbia, Italy) and dedicated EDGE Software (version 1.6.0.0);
- Charpy impact tests conducted on U-notched specimens using a ZWICK/ROELL HIT50P (ZwickRoell, Ulm, Germany) impact hammer with an impact energy of 50 J (according to the [57]);
- Hardness tests with a Mitutoyo HR-530 (Mitutoyo, Takatsu-ku, Japan) hardness tester (according to the [58])
- Tensile tests on a ZWICK/ROELL Z150 (ZwickRoell, Ulm, Germany) tensile testing machine (according to the [59]).

The analysis of austenite content was carried out using the X-ray diffraction method. The measurements were made with the Theta-Theta Edge diffractometer from GNR Analytical Instruments Group (G.N.R. S.r.l, Agrate Conturbia, Italy). The lamp was 4 W and a wavelength was *Kα λ* = 0.15418 nm. The diffractometer has a vertical goniometer with a *ψ* angle range from −45° to +45° and a 2*θ* angle range from 45° to 170°. The signal recording in each step of the detector lasted 9 s. The phenomenon of diffraction, i.e., the deflection of X-ray radiation on the crystal lattice, is described by the Bragg formula, which states that the condition for diffraction is that the integral multiple n of the wavelength *λ* incident on the crystal is equal to twice the distance between the crystallographic planes *d_hkl_* giving the diffraction effect and the sine of the angle of incidence of the radiation beam *Θ* on this crystallographic plane in accordance with Formula (1):

*n λ* = 2*d_hkl_*sin*Θ* (1)

When the angle of reflection is equal to the angle of incidence and at the same time the waves reflected from the successive crystallographic planes are in phase, the effect of the amplification of the beam diffracted on the crystal, called diffraction, will occur. By performing a diffraction experiment using the X-ray radiation of a known wavelength *λ* and measuring the angle *Θ* at which the amplification of the radiation beam occurs, the value of *d_hkl_*, which is characteristic for a specific crystal structure, can be calculated.

**Figure 2:**
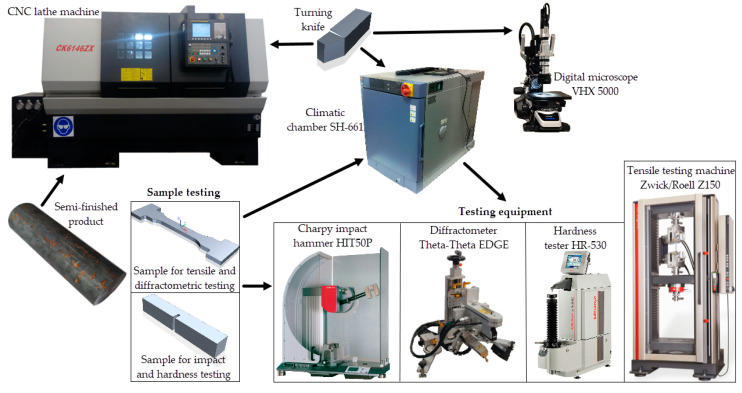
Experimental setup.

In addition, the abrasive wear resistance of the cryogenic-treated and non-cryogenic-treated tools was evaluated based on practical machining tests, rather than via standard abrasion testing. A scheme of the experimental setup is shown in Figure 2.

Test samples were made of molybdenum high-speed tool steel, whose designations according to various standards, chemical composition, and selected properties are listed in Table 1.

**Figure 3:**
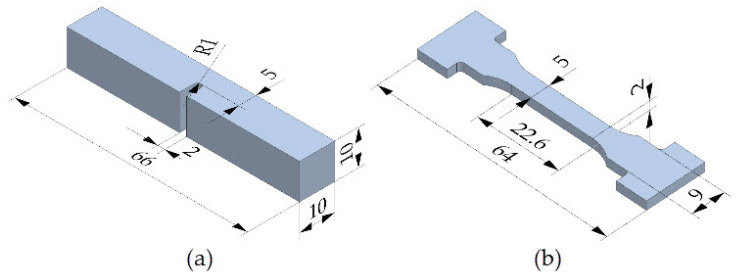
Samples used for (**a**) hardness and impact strength tests; (**b**) retained austenite measurement and tensile strength tests.

Two types of samples were used for testing mechanical properties and retained austenite concentration:

- Hardness and impact strength tests were conducted using U-notched specimens with a square cross-section of 10 × 10 mm, which were made in accordance with [57] and are shown in Figure 3a;
- Retained austenite measurements and peel tests were conducted using proportional specimens cut from a 2 mm thick flat bar in compliance with [59], which are shown in Figure 3b.

Twelve samples of both types were fabricated, with half of the samples of each type subjected to cryogenic treatment. Prior to destructive testing, the samples were examined for hardness and retained austenite content. Three measurements of hardness and retained austenite concentration were made for each sample.

**Figure 4:**
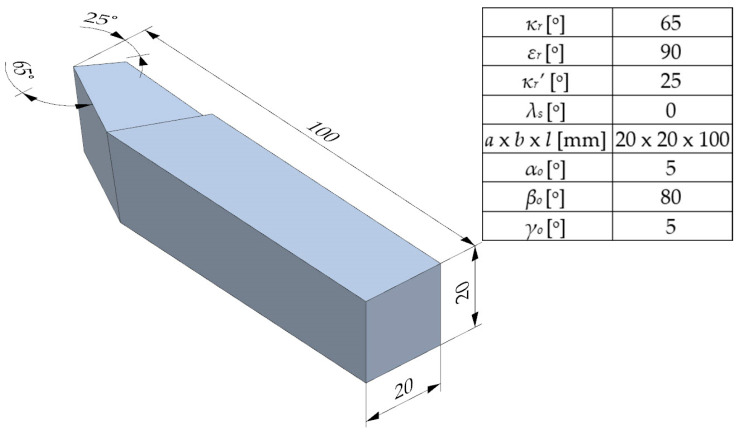
Schematic design and basic geometric parameters of ISO 1 R tool that was used for cutting edge life tests.

In order to assess the abrasive wear resistance, two cutting tools of the ISO 1 R type were made from an M2 steel bar with a cross-section of 20 × 20 mm. The tools and their basic geometrical parameters are shown in Figure 4. One of these tools, together with the samples described earlier, was subjected to cyclic cryogenic treatment.

For both tools, i.e., with cryogenic treatment and without it, the cutting edge wear was assessed via turning a shaft with an initial diameter of *d* = 60 mm, made of C45 steel, whose designations according to various standards, chemical composition, and selected properties are listed in Table 2.

It was decided that cutting edge wear would be assessed via turning because it allowed relatively easy measurement of direct wear indicators with a Keyence VHX 5000 (Keyence, Osaka, Japan) digital microscope. Machining tests were performed on a CNC CK6146ZX lathe from MTP, using the following machining parameters:

- Depth of cut: *a_p_* = 1 mm;
- Feed: *f* = 0.1 mm/rev;
- Cutting speed: *v_c_* = 25 m/min.

The assessment of wear and the measurement of wear indicators were carried out in compliance with the [61]. Most of the wear indicators specified in this standard were measured, while the tool wear was estimated based on two indicators:

- For the flank face: the tool flank wear indicator *VB_C_*;
- For the rake face: the tool tip wear indicator *KE*.

## 3. Results and Discussion

The study involved comparing retained austenite concentrations, selected mechanical properties, and wear resistance of the tools and samples made from M2 high-speed tool steel, with cryogenic treatment and without it. Results of individual tests are discussed in subsections that follow.

### 3.1. Retained Austenite Volume Fraction

**Figure 5:**
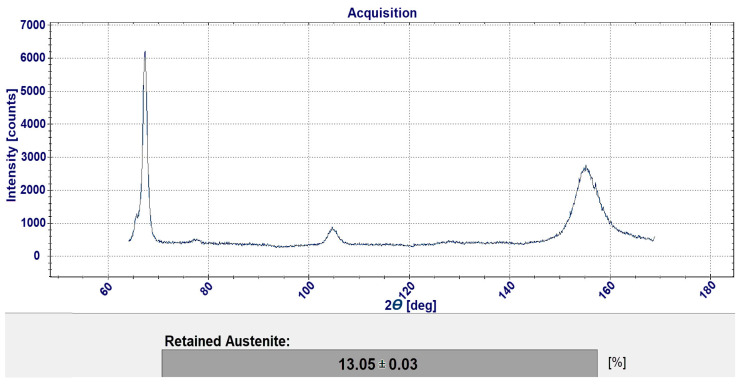
Example of measurement of retained austenite.

At the beginning of the study, it was decided that the samples would be subjected to a single cycle of shallow cryogenic treatment. The applied temperature range was due to the capabilities of the environmental chamber, which only allowed the temperature descent to −70 °C. Both the samples subjected to this treatment and the samples without this treatment were examined for retained austenite by the X-ray diffraction method. The diffractometer and the accompanying software allowed direct measurement and reading of the volume fraction of retained austenite. An example of a measurement result obtained by X-ray diffraction is shown in Figure 5.

**Figure 6:**
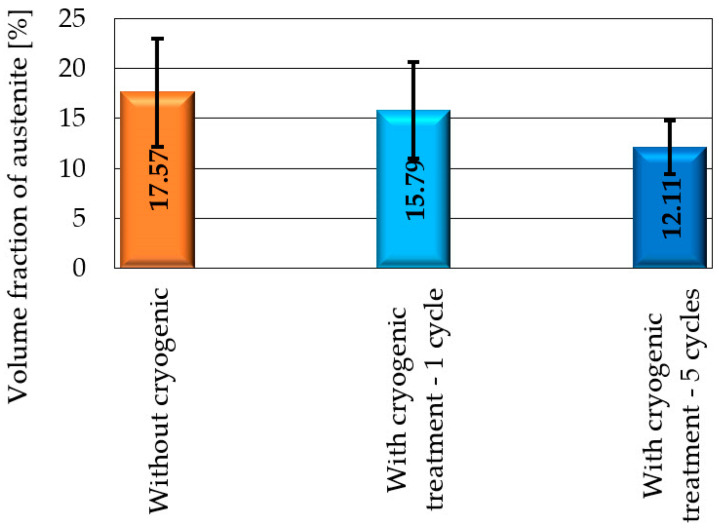
Retained austenite volume fraction.

The reduction in retained austenite obtained after a single cycle of cryogenic treatment was considered insufficient, so successive tests were carried out using several cycles of this treatment. Following these tests and taking into account the treatment time and the obtained austenite concentration reduction, it was decided that cryogenic treatment would be run in five cycles in further tests. A comparison of the retained austenite concentration results for the samples without cryogenic treatment and after one and five cryogenic treatment cycles is given in Figure 6.

In Figure 6, one can observe an approx. 1% decrease in retained austenite for the samples after one cycle of cryogenic treatment when compared to the samples without this treatment. In addition, the standard deviations of retained austenite concentration are so high that it is difficult to determine the significance of the differences in the results for both types of samples. It can therefore be assumed that the application of one cycle of cryogenic treatment practically did not reduce the retained austenite concentration. The volume fraction of retained austenite in the steel samples subjected to cyclic cryogenic treatment decreased by more than 5%. Compared to the non-cryogenic-treated samples, the retained austenite concentration in the samples subjected to cyclic cryogenic treatment decreased by over 30%. Also, the standard deviation of retained austenite for the samples subjected to five cycles of cryogenic treatment was significantly lower than that obtained for the samples without this treatment. This indicates a beneficial effect of the cryogenic treatment on material structure homogenisation, thus confirming the findings of previous studies mentioned in the Introduction.

In tests and measurements that followed, focus was put on comparing the results for M2 steel without cryogenic treatment and after five cycles of this treatment, which is why in the remainder of the paper the authors refer to the cyclic treatment when using the term cryogenic treatment.

### 3.2. Hardness

**Figure 7:**
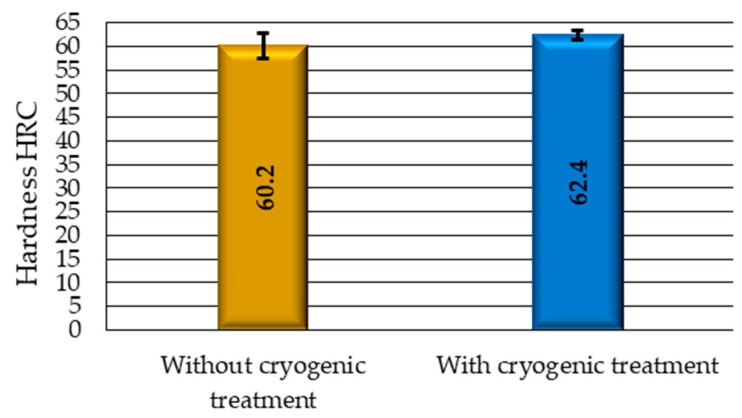
Results of hardness HRC.

In cryogenic processing, the low temperature causes retained austenite to transform into much harder martensite. In addition, carbides are precipitated as a result of the low temperature. These two processes should lead to increased hardness of the steel. A comparison of the HRC hardness obtained for the cryogenic-treated and non-cryogenic-treated samples is shown in Figure 7.

The results demonstrate that the cryogenic-treated steel exhibits an increase of more than 2 units in hardness on the HRC scale, which translates into an approximately 4% increase in their hardness compared to the material without cryogenic treatment. At the same time, like in the case of retained austenite, the standard deviation of HRC hardness for the cryogenic-treated samples is significantly smaller than for the samples without this treatment, which confirms the beneficial effect of cryogenic treatment on material structure homogeneity. It should be emphasised that the tool edge hardness is a key machinability indicator and directly affects the cutting tool life.

### 3.3. Impact Strength and Tensile Strength

**Figure 8:**
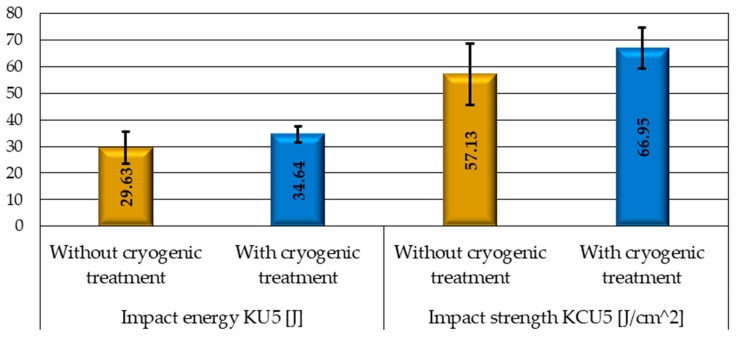
Results of Charpy impact tests.

**Figure 9:**
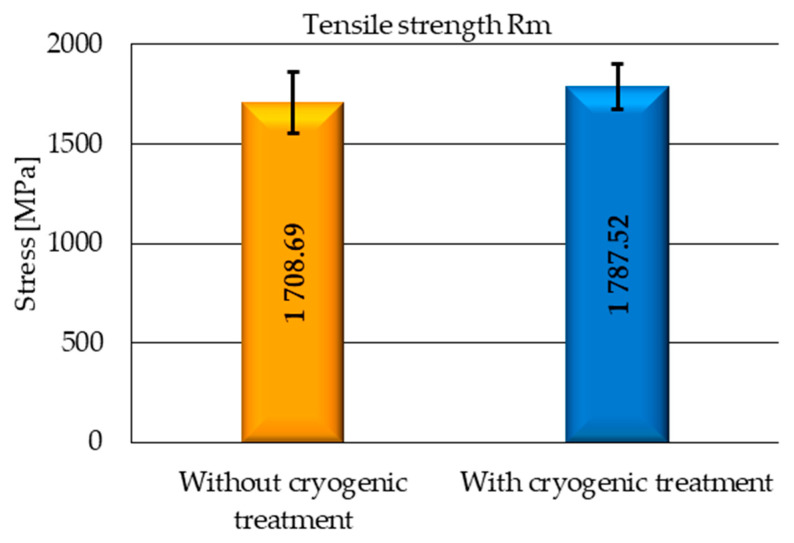
Tensile test results—tensile strength *R_m_*.

According to the literature, cryogenic treatment leads not only to transforming retained austenite into martensite, but it also causes carbide precipitation, which results in enhanced strength properties of steels. To determine the effect of cryogenic treatment on selected mechanical properties of M2 steel, the samples were subjected to Charpy impact tests (Figure 8) and tensile tests to evaluate the tensile strength *R_m_* (Figure 9).

Figure 8 shows the results of Charpy impact tests, revealing an approx. 17% increase in the impact strength of the cryogenic-treated samples compared to the samples without cryogenic treatment. On the other hand, an analysis of the tensile test results demonstrates that the increase in the tensile strength *R_m_* (Figure 9) of the cryogenic-treated samples is not as considerable as in the case of impact strength and only amounts to about 5%.

### 3.4. Abrasive Wear—Cutting Edge Life

**Figure 10:**
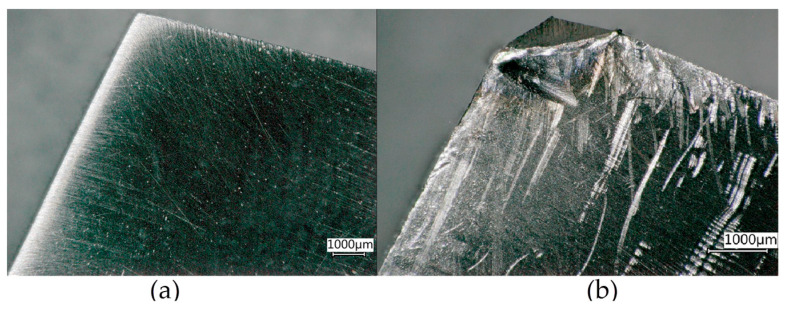
Images of an ISO 1 R tool edge without cryogenic treatment: (**a**) before treatment; (**b**) after treatment.

As mentioned previously, both the transformation of retained austenite into martensite and carbide precipitation lead to increased hardness of cryogenic-treated steels, which, for tool steels, should result in longer life (abrasive wear resistance) of the cutting edge. For comparing the life of tool edges with cryogenic treatment and without cryogenic treatment, turning tests were performed to determine tool edge life and produce wear curves. Images of an ISO 1 R tool edge before and after treatment (complete tool wear) are shown in Figure 10.

**Figure 11:**
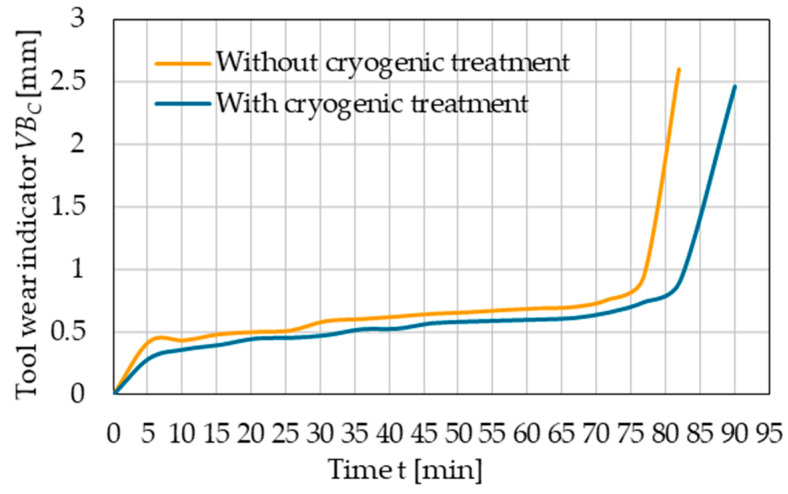
Tool wear indicator *VB_C_* versus machining time.

**Figure 12:**
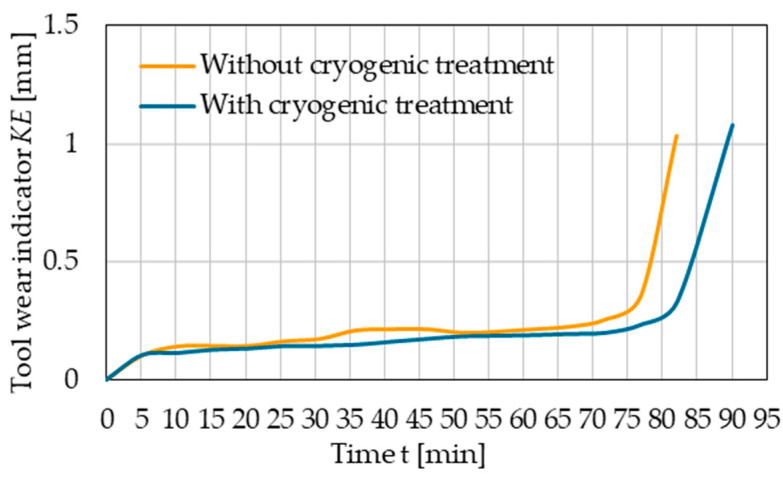
Tool wear indicator *KE* versus machining time.

The wear of the cryogenic-treated tool edges with cryogenic treatment and without this treatment was assessed by comparing the changes in the values of selected wear indicators as a function of time (wear curves). Figure 11 shows the results of the tool flank wear indicator *VB_C_*. For this indicator, the limit wear was determined on the flank face and was approximately *VB_C_* = 0.9 mm. As for the rake face, the limit wear was estimated using the tip wear indicator *KE*, and its value was found to be about *KE* = 0.35 mm (Figure 12).

A comparison of the wear curves and the time until the limit wear is reached demonstrates an approx. 10% increase in the life of the cryogenic-treated cutting tool edge compared to the tool without this treatment. For the case under study, this corresponds to an increase in the cutting edge life by about 8 min.

Based on the obtained results, regression equations and coefficient of determination values were also determined—Table 3. The high values of the *R*^2^ index indicate that tool wear indicators can be determined with a high degree of accuracy using the developed models.

## 4. Conclusions

The results of this study have demonstrated that the cyclic cryogenic treatment of M2 steel has led to a 30% reduction in retained austenite in this material. The transformation of austenite into martensite and probable carbide precipitation have improved the mechanical properties of this steel. Specifically,

- The tensile strength has increased by approximately 5%;
- The impact strength has increased by approximately 17%;
- The hardness has increased by approximately 4%;
- The cryogenic treatment has also improved the abrasive wear resistance of this material—the tool life has been prolonged by about 8 min (approx. 10% increase).

The differences in the results of the cryogenic-treated samples are smaller than those obtained for the samples without this treatment, which suggests that the alloy structure undergoes homogenisation as a result of the cryogenic treatment. It should also be highlighted that

- The cryogenic treatment is relatively inexpensive and can be carried out for a large number of tools simultaneously, which reduces the unit cost of this treatment;
- The changes in material structure resulting from cryogenic processing occur throughout the entire volume of the material, which is a definite benefit of this treatment and—for HSS cutting tools—constitutes an advantage over protective coating application;
- The cryogenic treatment of HSS lathe tools is not very practical these days due to the rare use of this type of tool. Nevertheless, the treatment can be widely applied to drilling tools, as they are still primarily made from HSS.

The authors plan to extend the research to other tool materials and other types of tools and machining operations. Additionally, deep cyclic cryogenic machining is being considered in the future.

## Figures and Tables

**Table 1:** Designations, chemical composition, and selected properties of M2 molybdenum high-speed tool steel [60].

Designation							
EN	W. no.	DIN		USA		Other	
HS6-5-2C	1.3343	HS6-5-2		M2 regular C		W6Mo5Cr4V2	
**Chemical composition %**							
C	Si max	Mn max	Cr	Mo	V	W	S max
0.86	-	-	3.80	4.70	1.70	5.90	-
0.94	0.45	0.40	4.50	5.20	2.10	6.70	0.035
**Properties**							
Elastic Modulus *E*		Compressive Yield Strength		Hardness, Rockwell *C*		Poisson’s Ratio	
190–210 GPa		3250 MPa		62–65		0.27–0.30	

**Table 2:** Denotations, chemical composition, and selected properties of C45 steel.

Denotation								
EN		W. no.				USA		
C45		1.0503				1045		
**Chemical composition %**								
C	Si	Mn	Cr max	Ni max	Mo max	Cu max	S max	P max
0.50	0.40	0.80	0.30	0.30	0.10	0.30	0.04	0.04
**Properties**								
Elastic modulus *E*		Tensile strength *R_m_*		Elongation *A_5_*	Upper yield strength *R_e_*		Brinell hardness HB	
190–205 GPa		560–850 MPa		14–17 %	275–490 MPa		≤229 HB	

**Table 3:** Regression equations for tool wear indicators.

Without Cryogenic Treatment		
*VB_C_*	y = 6 × 10^−10^ x^6^ 1 × 10^−7^ x^5^ + 7 × 10^−6^ x^4^ − 2 × 10^−4^x^3^ + 1 × 10^−5^ x^2^ + 0.0528x + 0.0474	*R*^2^ = 0.9747
*KE*	y = 2 × 10^−10^ x^6^ − 4 × 10^−8^ x^5^ + 3 × 10^−6^ x^4^ − 8× 10^−5^x^3^ + 6 × 10^−4^x^2^ + 0.0122x + 0.0115	*R*^2^ = 0.9801
**With Cryogenic Treatment**		
*VB_C_*	y = 2 × 10^−10^ x^6^ 4 × 10^−8^ x^5^ + 2 × 10^−6^ x^4^ 4 × 10^−5^x^3^ − 1.1 × 10^−3^x^2^ + 0.0468x + 0.0242	*R*^2^ = 0.9943
*KE*	y = 1 × 10^−6^x^6^ – 2 × 10^−8^x^5^ + 1 × 10^−6^x^4^ 3 × 10^−5^x^3^ − 1 × 10^−4^x^2^ + 0.014x + 0.0114	*R*^2^ = 0.9957

## Data Availability

The original contributions presented in the study are included in the article, further inquiries can be directed to the corresponding author.
